# Dexamethasone versus standard treatment for postoperative nausea and vomiting in gastrointestinal surgery: randomised controlled trial (DREAMS Trial)

**DOI:** 10.1136/bmj.j1455

**Published:** 2017-04-18

**Authors:** DREAMS Trial Collaborators and West Midlands Research Collaborative

## Abstract

**Objectives** To determine whether preoperative dexamethasone reduces postoperative vomiting in patients undergoing elective bowel surgery and whether it is associated with other measurable benefits during recovery from surgery, including quicker return to oral diet and reduced length of stay.

**Design** Pragmatic two arm parallel group randomised trial with blinded postoperative care and outcome assessment.

**Setting** 45 UK hospitals.

**Participants** 1350 patients aged 18 or over undergoing elective open or laparoscopic bowel surgery for malignant or benign pathology.

**Interventions** Addition of a single dose of 8 mg intravenous dexamethasone at induction of anaesthesia compared with standard care.

**Main outcome measures** Primary outcome: reported vomiting within 24 hours reported by patient or clinician. Secondary outcomes: vomiting with 72 and 120 hours reported by patient or clinician; use of antiemetics and postoperative nausea and vomiting at 24, 72, and 120 hours rated by patient; fatigue and quality of life at 120 hours or discharge and at 30 days; time to return to fluid and food intake; length of hospital stay; adverse events.

**Results** 1350 participants were recruited and randomly allocated to additional dexamethasone (n=674) or standard care (n=676) at induction of anaesthesia. Vomiting within 24 hours of surgery occurred in 172 (25.5%) participants in the dexamethasone arm and 223 (33.0%) allocated standard care (number needed to treat (NNT) 13, 95% confidence interval 5 to 22; P=0.003). Additional postoperative antiemetics were given (on demand) to 265 (39.3%) participants allocated dexamethasone and 351 (51.9%) allocated standard care (NNT 8, 5 to 11; P<0.001). Reduction in on demand antiemetics remained up to 72 hours. There was no increase in complications.

**Conclusions** Addition of a single dose of 8 mg intravenous dexamethasone at induction of anaesthesia significantly reduces both the incidence of postoperative nausea and vomiting at 24 hours and the need for rescue antiemetics for up to 72 hours in patients undergoing large and small bowel surgery, with no increase in adverse events.

**Trial registration** EudraCT (2010-022894-32) and ISRCTN (ISRCTN21973627).

## Introduction

Postoperative nausea and vomiting (PONV) are the most common complications after surgery, affecting more than 30% of patients,^1^ ^2^ and are reported by patients to be two of the five most undesirable outcomes.^3^ Evidence has been conflicting regarding type of surgery and risk of PONV, but a systematic review has shown that laparoscopic surgery and increasing duration of surgery are independent predictors of higher risk.^4^ There are many published studies reporting increased risk after urological or biliary tract surgery, but there is a relative paucity of data regarding small or large bowel surgery. A recent study has shown that even after introduction of an enhanced recovery protocol, nearly 35% of patients undergoing colonic resection required antiemetics on demand in the postoperative period,^5^ and so PONV remains a common adverse event even within a selected optimised population.^5^ PONV is related to anaesthetic use in many operations, but in bowel surgery, the bowel handling, resection, and associated intra-abdominal contamination promote an ileus of variable duration and has a major effect on PONV. PONV contributes to both delayed recovery and delayed discharge, and its prevention is of particular importance after gastrointestinal surgery because these patients often have poor preoperative nutrition, as highlighted by a UK nutrition survey in which 40% of patients with gastrointestinal disease were deemed at high risk of malnutrition on admission to hospital, significantly higher than the baseline for all hospital admissions.^6^

Dexamethasone is a potent corticosteroid that has been reported to influence patient and clinician based outcome measures of recovery.^7^ ^8^ It has been carefully assessed in the prevention of PONV in low and intermediate risk surgery and shown to have a substantial benefit. In a trial of over 4000 patients that assessed 64 different combinations of anaesthetic measures, dexamethasone effectively reduced PONV.^9^ Only 11% of patients, however, underwent abdominal procedures, of whom only a small fraction underwent bowel surgery.^9^ The study found that multiple interventions (use of more than one antiemetic) should be reserved for high risk patients. As outlined above, patients undergoing large or small bowel surgery have not historically been thought to be at high risk.

Dexamethasone is one of several drugs recommended for patients at moderate and high risk of PONV in the consensus guidelines from the Society for Ambulatory Anesthesia^10^ and is commonly advocated in protocols for enhanced recovery after gastrointestinal surgery (ERAS).^11^ ^12^ ^13^ Its value in bowel surgery, however, is unproved and its use is far from universal. Two single centre trials, totalling 100 patients undergoing bowel surgery, reported no benefit of dexamethasone on PONV.^14^ ^15^ Before designing this trial, we surveyed six major colorectal units in the West Midlands region and found that only 25% of colorectal patients were receiving dexamethasone on induction of anaesthesia.

Prolonged use of steroids such as dexamethasone can have dangerous side effects, including an increased risk of wound infection and anastomotic leak, which adversely affect recovery from gastrointestinal surgery. A single dose, however, does not seem to be associated with this increased risk. Though a systematic review of single dose methylprednisolone in patients undergoing cardiac, general, or trauma surgery showed no significant increase in these adverse events,^16^ concerns among the surgical community might still be limiting its use during surgery of the large and small intestine.

The mechanism of action of dexamethasone is poorly understood, but it seems to be most effective when it is administered before the start of surgery, when it can also reduce surgery induced inflammation.^17^ A meta-analysis of patients undergoing thyroidectomy found that dexamethasone at 8-10 mg had the greatest effect in reducing PONV.^18^

The DREAMS trial examined whether preoperative dexamethasone reduces postoperative vomiting in patients undergoing elective gastrointestinal surgery. Reduction of PONV in this group of patients could improve the surgical experience and also fast track recovery and decrease postoperative complications. In assessing whether multi-modal use of antiemetics in this cohort improves outcomes, we designed a pragmatic trial that would incorporate the use of one standard antiemetic with or without the addition of a single dose of 8 mg intravenous dexamethasone, a drug with proved antiemetic properties (in other groups of patients) but that has a different mechanism of action to the other commonly used antiemetics. If beneficial, dexamethasone could be a valuable addition for patients undergoing intestinal surgery.

## Methods

### Study design and eligibility

DREAMS is a pragmatic blinded multicentre randomised controlled trial of 1350 participants comparing the effects of a single dose of 8 mg intravenous dexamethasone against standard care on patient recovery after small and large bowel surgery. Though the anaesthetist knew the treatment allocation, patients and clinical staff involved in postoperative care and data collection were blinded to it. The trial included an internal pilot study of 150 participants to assess recruitment rates, the randomisation process, patient pathway, and data collection tools (outcomes from participants in the pilot phase are included in this main trial analysis and have not been analysed previously).

Eligible patients were adults (aged 18 and over) with ability to consent who were undergoing elective open or laparoscopic bowel surgery for malignant or benign pathology. Patients were approached at the first outpatient clinic, at the preoperative assessment clinic, or on admission for surgery. Once eligibility had been confirmed, patients gave informed consent either at the preoperative visit or on admission. Patients who were pregnant or who had gastrointestinal obstruction, diabetes, glaucoma, or active gastric ulceration confirmed by endoscopy were ineligible (all patients had blood glucose concentrations checked to exclude undiagnosed hyperglycaemia). Those with a known adverse reaction to dexamethasone or who were taking any systemic steroids (excluding steroid inhalers, suppositories, pessaries, eye drops, one-off local injections to a joint, or topical preparations) were excluded from the trial. Patients previously taking regular oral or intravenous steroids had to have stopped taking these drugs at least three months before trial entry to be eligible.

### Interventions

All patients underwent general anaesthesia and received a routine antiemetic (other than dexamethasone) preoperatively as standard care determined by the anaesthetist. The key specified standard of care was that a single dose of an antiemetic would be given before the start of surgery. The anaesthetic team were directed to obtain the treatment allocation from the trials unit after induction of anaesthesia and administration of their choice of antiemetic, thus ensuring allocations were concealed until standard care had been delivered. Those patients allocated to dexamethasone received 8 mg intravenous dexamethasone before the start of surgery; those allocated to control received nothing in addition to standard care. All antiemetics were administered before knife to skin and no further antiemetics were administered during the operation.

Postoperative antiemetics were administered at the request of the patient. The treating anaesthetists were not involved in postoperative care other than in exceptional circumstances for medical emergencies in the acute postoperative period. Dexamethasone was not prescribed within the first 24 hours postoperatively for participants in either arm.

### Randomisation and blinding

Participants were randomly assigned 1:1 between dexamethasone and standard care. Allocation was made by a web based central randomisation service at the University of Birmingham clinical trials unit, with telephone backup. The system used a computerised minimisation procedure to reduce the risk of chance imbalances in important stratification variables of sex, smoking status (yes, no), type of surgery (open, laparoscopic), intended postoperative analgesia (patient controlled analgesia (PCA), epidural), American Society of Anesthesiologists (ASA) grade (1, 2, 3, 4, 5), and whether participants were within the enhanced recovery after surgery (ERAS) pathway (yes, no). The record of administration of dexamethasone was coded only on the trial specific intraoperative form by the anaesthetist, and not in the routine anaesthetic record within the medical notes, to ensure staff involved in postoperative care remained blinded to the treatment allocation. Each form was placed in a sealed envelope and sent to the trials unit. We aimed to minimise the impact of the anaesthetist being aware of the allocation by timing of randomisation to be after administration of the standard antiemetic, ensuring that no paperwork existed in the patient notes and that anaesthetists were clearly informed that they should not reveal the allocation to patients or clinical staff. The same anaesthetist would only exceptionally be involved in postoperative care, and the possibility that they would introduce a bias in postoperative antiemetic administration was judged minimal.

### Outcome measures

The primary outcome measure was any vomiting within 24 hours postoperatively, defined as episodes of expulsion of gastric content.^19^ To ensure that no episodes of vomiting were excluded from the analysis, patients who had vomiting episodes reported by either themselves or by staff were deemed to have experienced vomiting.

Secondary outcome measures included the number of episodes of vomiting postoperatively (with an interval of five minutes defining separate episodes), the use of postoperative antiemetics, severity of postoperative nausea and vomiting (measured with the PONV intensity scale^20^), fatigue (measured with the FACIT-F (functional assessment of chronic illness-fatigue) questionnaire^21^), time to toleration of oral diet, length of hospital stay, and health related quality of life (measured with EQ-5D-3L^22^ ^23^). Nausea, vomiting, and the use of antiemetics were measured during the 0-24 hour, 25-72 hour, and 73-120 hour postoperative periods; fatigue and health related quality of life measures were recorded on discharge. Outcomes are reported at all time points when they were measured. Participants were seen 30 days postoperatively and assessments made of wound and chest infections and other complications; attempts were made by telephone to contact participants who did not attend.

### Statistical analysis

The sample size was chosen to detect a 24% proportional reduction in the number of participants experiencing vomiting in the first 24 hours after surgery (corresponding to a reduction from 37% to 28% based on a large factorial trial^9^). The initial sample size of 950 patients provided 80% power to detect this difference (at a two tailed significance level of 0.05, allowing for 10% loss to follow-up). The independent data monitoring committee met three times and reviewed interim analyses at time points when 365, 700, and 1170 participants had been recruited. Because of faster than expected recruitment, at their second meeting the committee advised increasing power to 90%, raising the final target sample size to 1320. At the same time, the committee also clarified that the primary outcome should include episodes of vomiting reported by both patients and clinicians recorded in patient notes to ensure that all episodes were captured. To investigate the impact of their decision, we also report (as a sensitivity analysis) the effect of treatment separately for vomiting episodes documented by clinicians and reported by patients.

Participants were analysed according to their allocation regardless of the treatment received. We calculated proportions of participants in each group experiencing vomiting, nausea, receiving on demand antiemetics, and returning to oral diet in each group and described treatment effects with risk ratios and numbers needed to treat for the 0-24, 25-72, and 73-120 hour postoperative periods. The number of on demand antiemetics given postoperatively, PONV, fatigue, and quality of life scores were summarised with means and standard deviations. All treatment effects are presented with 95% confidence intervals. For comparisons of dexamethasone versus no dexamethasone, we used *t* tests for continuous variables, χ^2^ tests for categorical variables, and χ^2^ tests for trend for ordinal variables. In the primary analysis we assumed that patients who had been discharged or had missing data were no longer experiencing vomiting or using additional antiemetics and explored the impact of this assumption in a sensitivity analysis. We assessed the consistency of the primary treatment effect across the stratification variables using Cochran’s test for heterogeneity for categorical variables and the Mantel-Haenszel tests of association for ordinal variables. Analysis of nausea and vomiting scores, fatigue, and quality of life reported by patients was restricted to those for whom data were available, and analyses of change from baseline were undertaken as a sensitivity analysis when possible. We analysed data on length of stay using log rank methods. All reported P values are two tailed, and P<0.05 was considered significant. All analyses were performed with SAS 9.4 (SAS Institute) or Stata V13.0 (StataCorp).

### Patient involvement

The trial was discussed and presented to patient advocates (at Independent Cancer Patients’ Voice), and the conduct of the trial was overseen by a patient advocate on the management team (Lindy Berkman), with specific input to the patient information sheet, consent, primary endpoint, and patient follow-up of the trial. A summary of the findings of the trial has been provided to the recruiting centres for dissemination to the trial participants.

## Results

### Patients’ characteristics

From July 2011 to January 2014, we recruited 1350 participants from 45 UK sites; 674 were randomly allocated to receive 8 mg intravenous dexamethasone at induction and 676 to standard induction without dexamethasone (fig 1). An additional 1544 participants were screened for inclusion but did not meet the criteria (728 (47.2%) were ineligible, 393 declined to participate (25.4%), study staff were not available to recruit 285 (18.5%), and reasons were unknown for 138 (8.9%)), and seven gave consent but not randomised. Five participants did not receive dexamethasone as allocated, and 10 participants from the control arm received dexamethasone.

**Figure f1:**
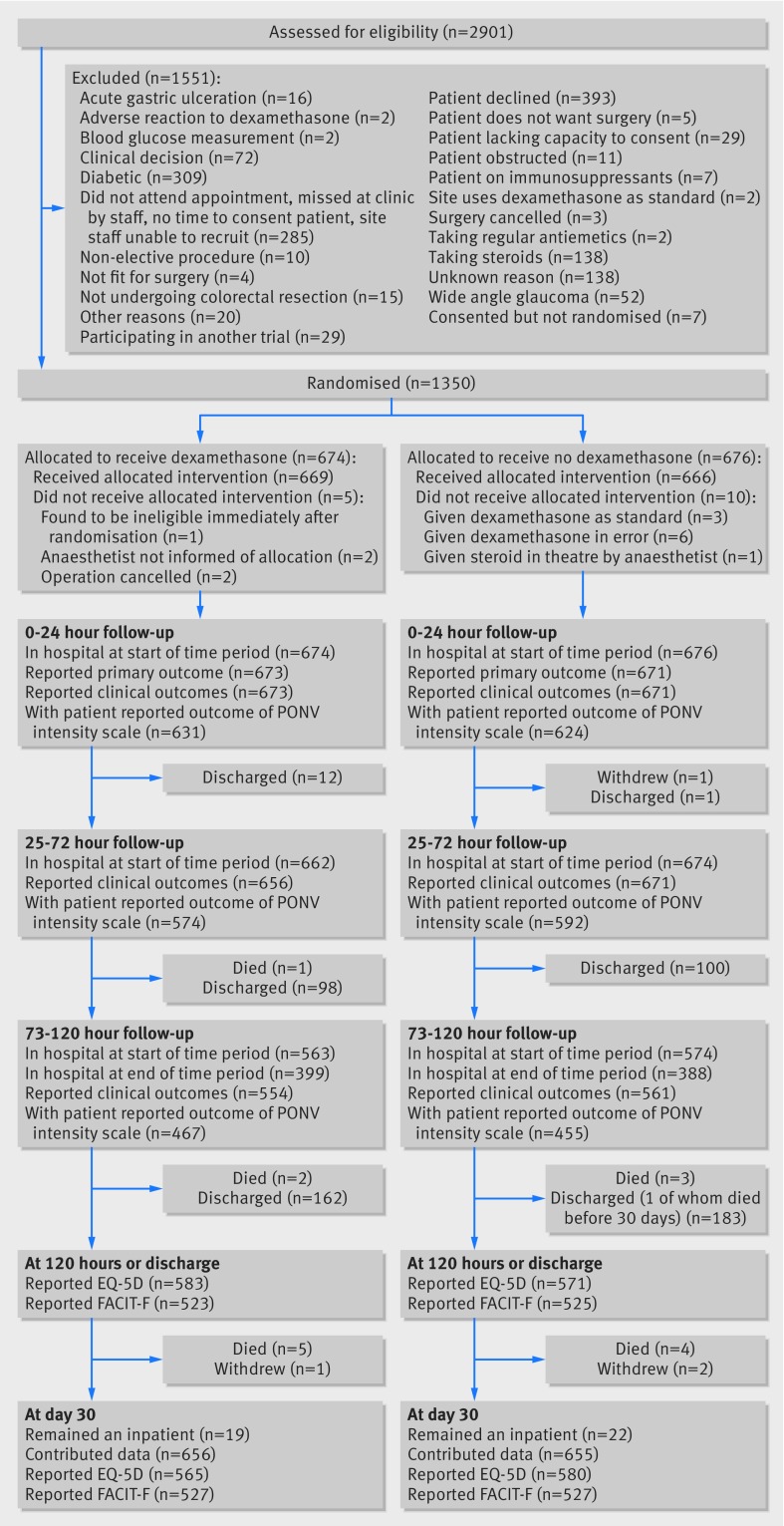
**Fig 1** Flow of patients undergoing gastrointestinal surgery through trial of dexamethasone versus standard treatment for postoperative nausea and vomiting

Patient demographics (table 1) and anaesthetic agents, type/duration of surgery, and planned postoperative analgesia (table 2) were similarly distributed across treatment groups. Most participants underwent major bowel surgery (mean duration 226 mins), and 1321 (97.9%) had a bowel resection and/or stoma formation/closure. A high proportion (856, 63%) underwent a laparoscopic or laparoscopic assisted procedure. Standard preoperative antiemetics were given to 1298 (96%) participants before randomisation.

**Table 1 tbl1:** Baseline characteristics of trial participants undergoing gastrointestinal surgery according to allocation to preoperative dexamethasone or standard care (no dexamethasone). Figures are numbers (percentage) of patients unless stated otherwise

	Dexamethasone (n=674)	No dexamethasone (n=676)	Total (n=1350)
Age (years) at randomisation:			
<50	91 (13.5)	97 (14.4)	188 (13.9)
50-59	128 (19.0)	120 (17.8)	248 (18.4)
60-69	214 (31.8)	223 (33.0)	437 (32.4)
70-79	189 (28.0)	172 (25.4)	361 (26.7)
≥80	52 (7.7)	64 (9.5)	116 (8.6)
Mean (SD)	63.6 (13.4)	63.4 (13.5)	63.5 (13.4)
Range	19-93	18-90	18-93
Sex:			
Women	283 (42.0)	284 (42.0)	567 (42.0)
Men	391 (58.0)	392 (58.0)	783 (58.0)
Smoking status:			
Non-smoker	574 (85.2)	576 (85.2)	1150 (85.2)
Smoker	100 (14.8)	100 (14.8)	200 (14.8)
Pack years (smokers only):			
Mean (SD)	27.4 (17.6)	22.9 (19.8)	25.1 (18.8)
Range	0.5-80	0.04-114	0.04-114
ASA grade:			
P1 normal healthy patient	157 (23.3)	155 (22.9)	312 (23.1)
P2 mild systemic disease	402 (59.6)	405 (59.9)	807 (59.8)
P3 severe systemic disease	113 (16.8)	113 (16.7)	226 (16.7)
P4 severe life threatening disease	2 (0.3)	3 (0.4)	5 (0.4)

ASA=American Society of Anesthiologists.

**Table 2 tbl2:** Characteristics of surgery, anaesthesia, and antiemetics in patients undergoing gastrointestinal surgery according to allocation to receipt of preoperative dexamethasone or standard care (no dexamethasone). Figures are numbers (percentage) of patients unless stated otherwise

	Dexamethasone (n=674)	No dexamethasone (n=676)	Total (n=1350)
Abdominal access:			
Laparoscopic	429 (63.7)	427 (63.2)	856 (63.4)
Open	245 (36.3)	249 (37.8)	494 (36.6)
Enhanced recovery after surgery programme:			
No	54 (8.0)	53 (7.8)	107 (7.9)
Yes	611 (90.7)	615 (91.0)	1226 (90.8)
Not known	9 (1.3)	8 (1.2)	17 (1.3)
Duration of anaesthesia (mins):			
<60	5 (0.7)	10 (1.5)	15 (1.1)
60-119	55 (8.2)	56 (8.3)	111 (8.2)
120-239	333 (49.4)	312 (46.2)	645 (47.8)
≥240	277 (41.1)	294 (43.5)	571 (42.3)
Missing	4 (0.6)	4 (0.6)	8 (0.6)
Mean (SD)	226 (99)	226 (108)	226 (103)
Range	45-660	15-1545	15-1545
Type of surgery:			
Stoma formation	8 (1.2)	9 (1.3)	17 (1.3)
Stoma reversal	66 (9.8)	76 (11.2)	142 (10.5)
Small bowel surgery	7 (1.0)	9 (1.3)	16 (1.2)
Right colon resection	150 (22.3)	153 (22.6)	303 (22.4)
Left/sigmoid colon resection	122 (18.1)	99 (14.6)	221 (16.4)
Subtotal/total colectomy	27 (4.0)	22 (3.3)	49 (3.6)
Rectal resection	276 (41.0)	297 (43.9)	573 (42.4)
Other	17 (2.5)	9 (1.3)	26 (1.9)
Missing	1 (0.2)	2 (0.3)	3 (0.2)
Intraoperative anaesthetic agents used			
Induction agent received	669	672	1341
Propofol	651 (97.3)	650 (96.7)	1301 (97.0)
Other	18 (2.7)	22 (3.3)	40 (3.0)
Maintenance agent received	667	666	1333
Volatile agent	372 (55.8)	375 (56.3)	747 (56.0)
Volatile agent + remifentanil	213 (31.9)	192 (28.8)	405 (30.4)
Other	82 (12.3)	99 (14.9)	181 (13.6)
Reversal agent received	371	399	770
Neostigmine + glycopyrolate	337 (90.8)	359 (90.0)	696 (90.4)
Other	34 (9.2)	40 (10.0)	74 (9.6)
Intraoperative opioids used	601 (89.2)	594 (87.9)	1195 (88.5)
One opioid received	441	435	876
Fentanyl	215	215	430
Morphine	126	132	258
Other	100	88	188
Two opioids received	152	147	299
Fentanyl + morphine	76	79	155
Fentanyl + remifentanil	23	19	42
Other	53	49	102
Three opioids received	8	12	20
Fentanyl + morphine + remifentanil	5	6	11
Other	3	6	9
Intraoperative antiemetics given	637 (94.5)	661 (97.8)	1298 (96.1)
One antiemetic	582	526	1108
Ondansetron	524	468	992
Cyclizine	36	34	70
Other	22	24	46
Two antiemetics	55	127	182
Cyclizine + ondansetron	41	98	139
Ondansetron + metoclopramide	11	12	23
Other	3	17	20
Three antiemetics	0	7	7
No of standard antiemetics	674	676	1350
Mean (SD)	1.03 (0.37)	1.18 (0.47)	1.11 (0.43)
Postoperative analgesia			
Epidural	307 (45.6)	308 (45.6)	615 (45.6)
Patient controlled	238 (35.3)	238 (35.2)	476 (35.3)
Not known	68 (10.1)	70 (10.4)	138 (10.2)
Other	50 (7.4)	49 (7.3)	99 (7.3)
None	11 (1.6)	11 (1.6)	22 (1.6)

Contrary to the protocol, 55 (8%) participants in the dexamethasone arm and 134 (20%) in the standard care arm received more than one routine antiemetic preoperatively. The types of antiemetics used were comparable between the two arms (table 2).

One participant was found to be ineligible on day 1 of the study and withdrawn from follow-up. Follow-up data were available on 1344 (99.6%) for assessments at 24 hours (primary outcome) and on 1327 (98.3%) at 72 hours, and 1115 (82.6%) at 120 hours (secondary outcomes). Figure 1 shows the number of patients who returned patient reported outcome assessments. Contact was made with 1311 patients at 30 days to assess postoperative complications.

### Postoperative vomiting

Overall, 395 (29.3%) participants experienced the primary outcome of postoperative vomiting within 24 hours of surgery. Significantly fewer did so in the dexamethasone arm (n=172, 25.5%) than in the standard care arm (n=223, 33.2%) (risk ratio 0.77, 95% confidence interval 0.65 to 0.92; P=0.003) (fig 2). This means that 13 (95% confidence interval 5 to 22) patients would need to be given dexamethasone preoperatively to avoid one patient experiencing vomiting in the first 24 hours. Of the patients experiencing the primary outcome, 251 (63.5%) had vomiting episodes noted by both clinicians and patients, 119 (30.1%) by patients only, and 25 (6.3%) by clinicians only. The treatment effect was of similar magnitude in episodes recorded by clinicians (0.77, 0.62 to 0.95; P=0.02) or by patients (0.75. 0.63 to 0.89; P=0.001).

**Figure f2:**
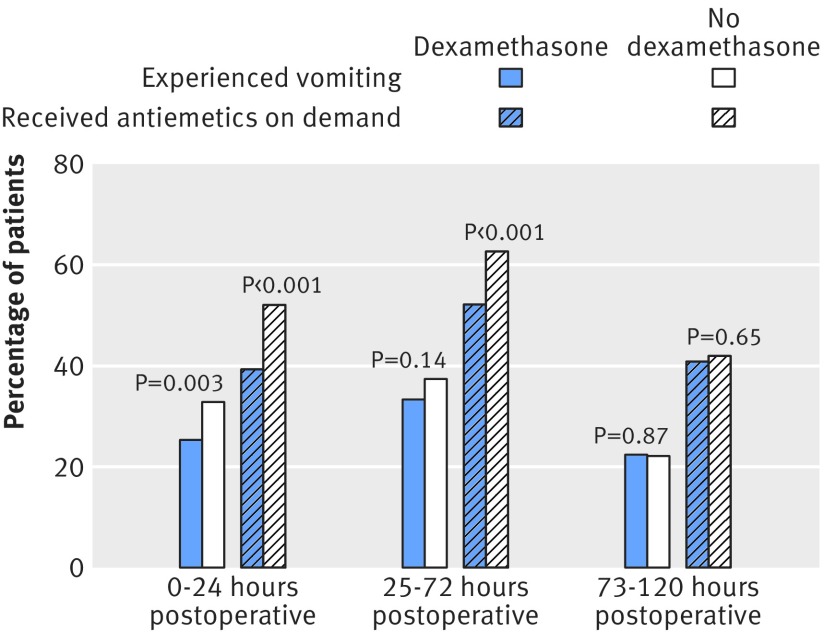
**Fig 2** Frequency of vomiting and use of antiemetics. Primary outcome is comparison of vomiting in 0-24 hours

Differences between episodes of vomiting were not significant in the period 25-72 hour after surgery (227 (33.7%) in the dexamethasone arm *v* 254 (37.6%) in the control arm; risk ratio 0.90, 95% confidence interval 0.78 to 1.03; P=0.14) and disappeared by 73-120 hours (152 (22.6%) *v* 150 (22.2%); 1.02, 0.83 to 1.24; P=0.87). Findings were similar when we excluded patients who had been discharged at the second and third time points and those with missing data. There was no evidence that the reduction in vomiting within 24 hours with dexamethasone differed in planned subgroup analyses according to type of surgery (P=0.91), whether the patient was assigned to an enhanced recovery pathway (P=0.51), smoking (P=0.68), ASA grade (P=0.79), postoperative pain relief (P=0.39), or sex (P=0.78) (fig 3).

**Figure f3:**
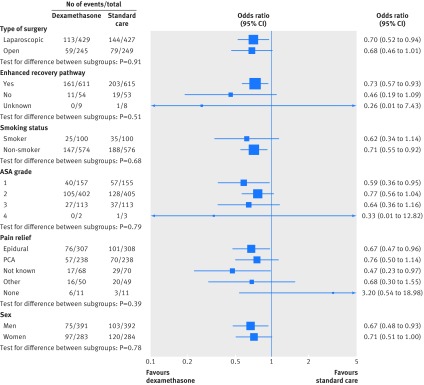
**Fig 3** Reduction in vomiting after gastrointestinal surgery within 24 hours (primary outcome) by subgroups according to allocation to preoperative dexamethasone or standard care

### On demand postoperative antiemetics

Postoperative antiemetics were used less by participants allocated dexamethasone for up to three days after surgery (fig 2). In the first 24 hours after surgery, they were given to 265 (39.3%) participants allocated dexamethasone and 351 (51.9%) participants allocated standard care (risk ratio 0.76, 95% confidence interval 0.67 to 0.85; P<0.001), such that giving eight (5 to 11) patients dexamethasone intraoperatively avoided one additional patient requiring antiemetics (for symptoms) in the first 24 hours. From 25 to 72 hours, antiemetics were given to 353 (52.4%) in the dexamethasone group and 425 (62.9%) on standard care (0.83, 0.76 to 0.91; P<0.001), with a number needed to treat of 9 (5 to 14). The mean number of doses and the number of antiemetics given were also lower across both these time periods in the dexamethasone group (table 2). Rates of use were similar in both groups from 73 to 120 hours, antiemetics being given to 276 (40.9%) participants allocated dexamethasone and 285 (42.2%) allocated standard care (0.97, 0.86 to 1.10; P=0.65). Findings were similar when we excluded patients who had been discharged at the second and third time points and those with missing data.

### Patient reported outcomes for PONV

The PONV intensity scale classifies patients as experiencing clinically important PONV based on their self rated frequency of vomiting and the intensity, pattern, and duration of nausea (patients who vomit three or more times, or patients who rate their nausea as intense, constant, and long lasting on the scoring system). Differences were seen at 24 hours after surgery (table 3): 54 (8.6%) participants had clinically important PONV in the dexamethasone arm compared with 79 (12.7%) on standard care (risk ratio 0.68, 95% confidence interval 0.49 to 0.94; P=0.02). This means that 25 (95% confidence interval 14 to 143) patients would need to be given dexamethasone preoperatively to avoid one patient experiencing self rated clinically important PONV in the first 24 hours. No differences were observed at 72 hours (1.06, 0.82 to 1.38; P=0.64) or 120 hours (1.00, 0.74 to 1.35; P=0.99). Differences were driven by differences in frequency of vomiting and nausea rather than difference in severity according to the VAS scoring (table 3 and fig A in appendix).

**Table 3 tbl3:** Patient reported outcomes for nausea and vomiting by time after gastrointestinal surgery according to allocation to preoperative dexamethasone or standard care (no dexamethasone). Figures are numbers (percentage) of patients unless stated otherwise with risk statistics and differences

	Dexamethasone	No dexamethasone	Risk ratio (95% CI)	Difference in risk (%) or means (95% CI)	P value
**24 hours**					
Clinically important PONV^*^	54/631 (9)	79/624 (13)	0.68 (0.49 to 0.94)	−4.0 (−7.5 to −0.7)	0.02
Patient reported vomiting/retching	158/652 (24)	212/652 (33)	0.75 (0.63 to 0.89)	−8.3 (−13.2 to −3.4)	0.001
Patient reported nausea	262/650 (40)	324/650 (50)	0.81 (0.72 to 0.91)	−9.5 (−14.9 to −4.2)	<0.001
Mean (SD) intensity (VAS scale^†^)	37.8 (26.6), n=251	41.7 (28.0), n=304	—	−3.9 (−8.5 to 0.7)	0.09
Return to oral diet:					
Any	654/673 (97)	644/672 (96)	1.01 (0.99 to 1.03)	1.3 (−0.6 to 3.3)	0.18
Fluids only	234/673 (35)	284/672 (42)	0.82 (0.72 to 0.94)	−7.5 (−12.7 to −2.3)	0.005
Diet and fluids	419/673 (62)	357/672 (53)	1.17 (1.07 to 1.29)	9.1 (3.9 to 14.4)	<0.001
Postoperative antiemetics given	265/674 (39)	351/676 (52)	0.76 (0.67 to 0.85)	−12.6 (−17.9 to −7.3)	<0.001
Mean (SD) No of types/patient	0.54 (0.76), n=672	0.78 (0.88), n=673	—	−0.23 (−0.32 to −0.14)	<0.001
Mean (SD) No of doses/patient	0.77 (1.25), n=670	1.07 (1.41), n=671	—	−0.31 (−0.45 to −0.17)	<0.001
**72 hours**					
Clinically important PONV^*^	96/574 (17)	93/592 (16)	1.06 (0.82 to 1.38)	1.0 −3.2 to 5.2)	0.64
Patient reported vomiting/retching	194/612 (32)	209/616 (34)	0.93 (0.80 to 1.10)	−2.2 (−7.5 to 3.0)	0.41
Patient reported nausea	324/613 (53)	349/616 (57)	0.93 (0.84 to 1.03)	−3.8 (−9.4 to 1.8)	0.18
Mean (SD) intensity (VAS scale^†^)	43.8 (29.1), n=298	44.5 (28.4), n=324	—	−0.7 (−5.2 to 3.9)	0.77
Return to oral diet					
Any	649/658 (99)	664/672 (99)	1.00 (0.99 to 1.01)	−0.2 (−1.4 to 1.0)	0.77
Fluids only	120 /658 (18)	128/672 (19)	0.96 (0.76 to 1.20)	−0.8 (−5.0 to 3.4)	0.70
Diet and fluids	527 /658 (80)	532/672 (79)	1.01 (0.96 to 1.07)	0.9 (−3.4 to 5.3)	0.68
Postoperative antiemetics given	353/674 (52)	425/676 (63)	0.83 (0.76 to 0.91)	−10.5 (−15.7 to −5.3)	<0.001
Mean (SD) No of types/patient	0.80 (0.86), n=656	0.96 (0.89), n=669	—	−0.16 (−0.25 to −0.06)	0.001
Mean (SD) No of doses/patient	1.70 (2.45), n=653	2.06 (2.61), n=665	—	−0.37 (−0.64 to −0.09)	0.009
**120 hours**					
Clinically important PONV^*^	74/467 (16)	72/455 (16)	1.00 (0.74 to 1.35)	0 (−4.7 to 4.7)	0.99
Patient reported vomiting/retching	132/497 (27)	129/479 (27)	0.99 (0.80 to 1.21)	−0.4 (−5.9 to 5.2)	0.90
Patient reported nausea	224/495 (45)	205/474 (43)	1.05 (0.91 to 1.21)	2.0 (−4.3 to 8.3)	0.53
Mean (SD) intensity (VAS scale^†^)	41.9 (26.3), n=58	46.5 (32.5), n=48	—	−4.6 (−16.0 to 6.7)	0.42
Return to oral diet:					
Any	539/555 (97)	547/560 (98)	0.99 (0.98 to 1.01)	−0.6 (−2.4 to 1.3)	0.56
Fluids only	75/555 (14)	79/560 (14)	0.96 (0.71 to 1.28)	−0.6 (−4.6 to 3.5)	0.77
Diet and fluids	463/555 (83)	465/560 (83)	1.00 (0.95 to 1.06)	0.4 (−4.0 to 4.8)	0.86
Postoperative antiemetics given	276/674 (41)	285/676 (42)	0.97 (0.86 to 1.10)	−1.2 (−6.5 to 4.1)	0.65
Mean (SD) No of types/patient	0.78 (0.90), n=553	0.81 (0.94), n=557	—	−0.03 (−0.14 to 0.08)	0.58
Mean (SD) No of doses/patient	2.23 (3.70), n=553	2.30 (4.10), n=555	—	−0.07 (−0.53 to 0.39)	0.78

*PONV (postoperative nausea and vomiting) intensity scale.

†High scores indicate severe nausea.

### Patient recovery from surgery, quality of life, and fatigue

Nearly all patients had started fluids by 24 hours (1298/1350, 96.1%; table 3). Of those who had started fluids at 24 hours, significantly more of the patients in the dexamethasone arm had also started eating compared with those who received standard care (419/673 (62.3%) *v* 357/672 (53.1%); risk ratio 1.17 (95% confidence interval 1.07 to 1.29); P<0.001). By 72 hours the return to diet was similar in both groups.

There were no differences in the EQ-5D index score or visual analogue scale at 120 hours and 30 days (table 4) and no difference in fatigue as measured by the FACIT-F score. Length of stay was similar, with a median of six days in both arms (interquartile range 4-9 in the dexamethasone arm, 4-10 in the control arm), and a log rank analysis also showed no difference (hazard ratio 1.02, 95% confidence interval 0.90 to 1.14; P=0.79) (fig B in appendix).

**Table 4 tbl4:** Fatigue (measured by FACIT-F^21^) and health related quality of life (measured by EQ5D^22^) by time after gastrointestinal surgery according to allocation to preoperative dexamethasone or standard care (no dexamethasone). Figures are mean (SD) scores and differences in means

	Dexamethasone	No dexamethasone	Difference in means (95% CI)	P value
**Baseline**				
EQ5D	0.85 (0.19), n=636	0.83 (0.21), n=637	0.02 (0 to 0.05)	0.03
EQ5D (VAS scale)	75.7 (17.8), n=642	74.7 (18.3), n=640	1.1 (−0.9 to 3.0)	0.29
FACIT-F (total score)	129.2 (22.0), n=588	127.5 (23.9), n=598	1.7 (−0.9 to 4.3)	0.20
**Discharge or 120 hours**				
EQ5D	0.54 (0.31), n=568	0.52 (0.31), n=561	0.02 (−0.02 to 0.05)	0.41
EQ5D (VAS scale)	59.2 (22.7), n=583	59.6 (21.5), n=571	−0.4 (−3.0 to 2.1)	0.74
FACIT-F (total score)	103.0 (27.9), n=523	102.0 (27.5), n=525	1.0 (−2.3 to 4.4)	0.54
**30 days**				
EQ5D	0.74 (0.26), n=562	0.75 (0.24), n=575	−0.01 (−0.03 to 0.02)	0.69
EQ5D (VAS scale)	72.4 (18.7), n=565	72.4 (18.1), n=580	0.0 (−2.2 to 2.1)	0.98
FACIT-F (total score)	121.4 (25.2), n=527	120.4 (26.4), n=527	1.1 (−2.1 to 4.2)	0.50
**Change from baseline to discharge/120 hours**				
EQ5D	−0.32 (0.33), n=543	−0.31 (0.31), n=535	0.0 (−0.04 to 0.04)	0.88
EQ5D (VAS scale)	−16.04 (24.55), n=562	−15.25 (23.44), n=549	−0.8 (−3.6 to 2.0)	0.58
FACIT-F (total score)	−26.11 (28.47), n=477	−26.05 (28.23), n=484	−0.1 (−3.6 to 3.5)	0.97
**Change from baseline to 30 days**				
EQ5D	−0.11 (0.27), n=534	−0.09 (0.26), n=548	−0.02 (−0.05 to 0.01)	0.18
EQ5D (VAS scale)	−3.59 (20.99), n=541	−2.46 (21.57), n=556	−1.1 (−3.7 to 1.4)	0.38
FACIT-F (total score)	−8.51 (26.46), n=475	−7.20 (25.53), n=481	−1.3 (−4.6 to 2.0)	0.43

### Adverse events

Thirty participants died; 13 (1.9%) in the dexamethasone arm and 17 (2.5%) in the control arm (risk ratio 0.77, 95% confidence interval 0.38 to 1.57; P=0.47). Eight deaths in each group occurred within 30 days postoperatively. There were no significant differences in the targeted adverse events between study arms: there were 147 reported infection episodes among 136 patients within 30 days: 69 (10.2%) patients in the dexamethasone arm and 67 (9.9%) patients in the control arm (1.03, 0.75 to 1.42; P=0.84). This included 84 patients with superficial wound infections (43 (6.4%) dexamethasone *v* 41 (6.1%) control; 1.05, 0.70 to 1.59; P=0.81), 19 patients with urinary tract infections (11 (1.6%) *v* 8 (1.2%); 1.38, 0.56 to 3.41; P=0.48), and 22 patients with respiratory infections (7 (1.0%) *v* 15 (2.2%); 0.47, 0.19 to 1.14; P=0.09). There were 32 patients with anastomotic leaks within 30 days (11 (1.6%) dexamethasone *v* 21 (3.1%) control; 0.53, 0.26 to 1.08; P=0.08) and three patients with intra-abdominal abscesses (2 (0.3%) dexamethasone *v* 1 (0.1%) control; 2.01, 0.18 to 22.1; P=0.62). No patient in either arm developed new onset diabetes. No suspected unexpected serious adverse reactions were reported, and no serious adverse reactions were judged as definitely related to trial treatment. There were two cases of sepsis and two gastrointestinal bleeds judged as probably or possibly related to trial treatment, which were split evenly across study arms.

## Discussion

**Overview**

In this multicentre pragmatic, blinded, randomised controlled trial testing the use of intravenous dexamethasone for prophylaxis of PONV in patients undergoing small and large bowel surgery, we have shown, for the first time, that a single 8 mg dose of intravenous dexamethasone at induction significantly reduces the incidence of PONV, reduces the need for rescue antiemetics, and increases the speed of return to diet after surgery. Furthermore, we showed dexamethasone to be safe in these patients, with no increase in adverse events. This strongly supports the use of dexamethasone as an induction antiemetic for patients undergoing bowel surgery.

Guidelines on the management of PONV in gastrointestinal surgery focus on a system for scoring severity to stratify patients for antiemetic use and have not been widely implemented into clinical practice.^10^ ^24^ This could be because previous studies have restricted the mode of anaesthesia and opioid use. DREAMS is the first pragmatic trial of dexamethasone in bowel surgery, providing evidence for its use across various anaesthetic protocols and surgical procedures.

A systematic review of 22 large studies identifying predictors of PONV found that female sex, history of PONV, non-smokers, younger age, volatile anaesthetics, duration of anaesthesia, and postoperative opioid use were the strongest factors associated with PONV.^4^ These factors were equally balanced between both arms of this trial, and we saw no evidence of differences in the relative reduction of vomiting according to these predictors. While maintenance with propofol is known to reduce PONV, only 93 patients (7%) in the trial received it.

The mechanism of action of dexamethasone is likely to be multifactorial,^25^ encompassing central neurological effects^26^ as well as anti-inflammatory benefits. A trial measuring cytokine levels in peritoneal drain fluid after colorectal surgery found significantly reduced levels of IL-6, a potent pro-inflammatory cytokine produced by T cells and macrophages in patients given dexamethasone.^15^ Our trial shows that the benefit from dexamethasone in terms of not requiring on demand antiemetics lasts beyond 48 hours, suggesting that the mechanism is unlikely to be simply anti-inflammatory. Murphy and colleagues found better global quality of recovery (QoR-40) scores in patients given dexamethasone compared with placebo (P<0.001) they when assessed recovery after discharge,^27^ supporting the late effect reported in our study.

### Strengths and limitations

We undertook this trial using an efficient and pragmatic approach to deliver a large study to quickly obtain clinically informative findings. The study was developed through the West Midlands Trainee Research Collaborative, providing effective teams of surgeons and anaesthetists in each recruiting centre. This structure combined with the avoidance of a placebo had a positive effect on recruitment to the study, which was completed six months ahead of schedule. These features could provide a template for future efficient trials.

There are, however, some limitations to this pragmatic trial. The efficient design precluded the use of a placebo, which would have totally ensured patient care was not altered by knowledge of the treatment allocation. Instead we designed the trial such that the individuals delivering postoperative patient care were blinded to the treatment allocation. The only individuals aware of the allocation were the anaesthetists, who were requested to abide by the study protocol. Anaesthetists administered antiemetics over and above that required by the protocol in 189 (14%) participants, however, most commonly in those allocated to standard care. This could reflect the fact that the patients recruited to this trial were undergoing major surgery undertaken by multidisciplinary teams of clinicians, when decisions to change practice during procedures are common. Notably, any bias introduced by the increased use of antiemetics in the standard care arm would have tended to reduce the observed differences between the groups. While we cannot completely exclude the possibility that the lack of blinding of anaesthetics could influence postoperative prescribing, this is highly unlikely to have occurred as it the exception that patients in the study would have been managed postoperatively on hospital wards by the same anaesthetist.

Detailed analyses of patient reported outcomes beyond 24 hours are restricted to those who remained in hospital (fig 1) and so will tend to reflect those with slower operative recovery. There was no noted difference, however, in length of stay between the study arms, so this is unlikely to have affected the comparison between study groups, and our sensitivity analyses, which investigated the impact of assuming no vomiting or antiemetic use after discharge, did not change our findings. We based our primary analysis on combined episodes of vomiting reported by patients or clinicians on the advice of the data monitoring committee to ensure we captured all relevant episodes. We undertook sensitivity analyses restricted to either patient or clinician reports, and the magnitude and significance of the difference at 24 hours remained in all analyses (risk ratio 0.75 (95% confidence interval 0.63 to 0.89; P=0.001) for patient reports; 0.77 (0.62 to 0.95; P=0.02) for clinician reports). At 72 hours the difference in vomiting was stronger and significant for clinician reported episodes (0.83 (0.70 to 0.98; P=0.03) than for patient reported episodes (0.93 (0.79 to 1.10; P=0.39).

The optimal dose of dexamethasone required to enhance postoperative recovery has not been established. Smaller doses (2.5-5 mg) have been reported to be effective,^28^ ^29^ although a dose of 8 mg has previously been reported as optimal in the prevention of PONV.^30^ ^31^ ^32^ In the absence of significant side effects, a recommended dose of 8 mg would seem safe for these patients. More detailed dietary records could show the nutritional impacts of perioperative interventions and could add value to future studies investigating gastrointestinal function after surgery.

We have shown that dexamethasone is safe in patients without diabetes at high risk of septic complications such as anastomotic leak and wound infection, and this is consistent with findings from studies of less contaminated procedures.^16^ ^26^ ^32^ ^33^ In their systematic review Waldron and colleagues found that patients receiving dexamethasone had small but significant increases in blood glucose 24 hours after operation.^33^ Studies have shown that a significantly higher blood glucose is seen in patients with impaired blood glucose preoperatively.^34^ ^35^ We excluded this high risk group from our trial in keeping with current consensus guidelines.^10^

At present, guidelines on the management and prevention of PONV are perhaps overly complex^10^ ^36^ and so not widely adopted. Our trial of patients undergoing small and large bowel surgery provides a simple solution for a reduction in PONV. We have shown that addition of a single intravenous 8 mg dose of dexamethasone significantly reduces PONV at 24 hours, is safe to use, and should be incorporated into routine clinical practice for patients without diabetes undergoing elective small and large bowel surgery.

**What is already known on this topic**

Postoperative nausea and vomiting (PONV) are the most common complications after surgery, affecting more than 30% of patients. In patients undergoing bowel surgery PONV often contributes to delayed recovery and is especially important because these patients might also have impaired preoperative nutrition
Dexamethasone has been shown to have a substantial benefit in the prevention of PONV in patient undergoing low and intermediate risk surgery but has not been evaluated in those undergoing bowel surgery

**What this study adds**

In patients undergoing bowel surgery, a single dose of 8 mg intravenous dexamethasone at induction of anaesthesia (in addition to standard care) had no significant increase in adverse events and reduced episodes of vomiting and patient rated clinically important postoperative nausea and vomiting for 24 hours after surgery
Patients receiving of a prophylactic single dose of 8 mg of intravenous dexamethasone before surgery also required fewer postoperative antiemetics for up to 72 hours

## Web Extra

Extra material supplied by the author

## Notes

This trial was supported both in development and delivery by the Birmingham Surgical Trials Consortium and the Royal College of Surgeons and Rosetrees Clinical Research Initiative. Local support for the pilot phase was also provided by the Birmingham ECMC. 
**DREAMS collaborative group**
Writing committee chief investigator: R Ravikumar (University of Oxford). Trial management committee and writing committee: D Bartlett, DG Morton (University of Birmingham); L Berkman (independent patient advocate); H Bodenham-Chilton, JJ Deeks, K Handley, L Magill (University of Birmingham Clinical Trials Unit); E Hamilton (Shrewsbury and Telford Hospital NHS Trust); E Hepburn (Cheltenham General Hospital); M-J Hwang (Sandwell and West Birmingham Hospitals NHS Trust); N Mirza (New Cross Hospital, Wolverhampton), A Wilkey (University Hospital Birmingham NHS Foundation Trust). University of Birmingham Clinical Trials Unit: H Bodenham-Chilton, M le Breuilly L Magill (trial management); A Wilcockson (programming); K Handley, S Mehta, JJ Deeks (statistics). Trial steering committee: A Burtenshaw, W Hawkins (chair), M Voysey. Independent data monitoring committee: J Blazeby (chair), I Smith, D Stocken. Participating centres and investigators (*principal investigator at each centre): S Abbott, M Hwang, A Karim, D P Luke, D McArthur, P Mistry, J Richardson, H Youssef* (Birmingham Heartlands Hospital); K Ravi,* P B Goodfellow, R Gupta (Chesterfield Royal Hospital); H Joy* (City Hospital, Birmingham); N Eardley, C McFaul,* D Vimalachandran (Countess Of Chester Hospital); C Harmston,* P Froggatt (University Hospital Coventry, Walsgrave); P Krishnan, S Pathare, V Shanmugam, Y J Yiannakou* (Darlington Memorial Hospital); A Fawole, C Macklin, J Mcenhill, H Narula, T Riad, A Rose* (Dewsbury and District Hospital); J Chambers, C Ekere, M Hough, J Hull, H P Knight, M J Lamparelli,* M Lewis, M Pulletz, N Siddiqi, B Stubbs, K Subramanian, R Swanton (Dorset County Hospital); S Costigan, G O’Connell, P K Patel* (Furness General Hospital); A Ali, C Ang, M A S Chapman, U Shariff, C Thompson, A Williamson J Yates, H Youssef* (Good Hope Hospital); D J Leinhardt,* M Simenacz (Harrogate District Hospital); S Harris, P Loveless,Y Mohsen,* A Myers, A Prabhudesai, S Vanagov (Hillingdon Hospital); K Aryal,* R Lal, D Millican, I Panagiotopopoulou, K Shankar (James Paget Hospital); M Dube,* J Tansley (King’s Mill Hospital); J Hill* (Manchester Royal Infirmary); S Addison, R Church,* J Nath, S Valap (Manor Hospital); N Mirza* (New Cross Hospital); A Dhrampal, J Nortje, J Payne, K Sargen, C Speakman* (Norfolk and Norwich University Hospitals); J Deloughry, T Elkington, R Dennis,* S Ghosh, J Martin, M Stoker (Peterborough City Hospital); N Akerman, M Basheer, N Drury, C Parchment-Smith, R Sandhu, R Srinivasa, S Varma, S Walwyn, A Rose,* N Syed, S Walwyn (Pinderfields Hospital); J Bromilow, P Colling, H Reschreiter* (Poole General Hospital); A Senapati* (Queen Alexander Hospital Portsmouth); F Howse, L Tzouliadis; S Bach, A Brookes, C Cagigas, N Dimitriou, S Fallis, K Futaba, E Hamilton, E Hepburn, A Hill, A Izbal, K Leong, D Morton,* M Manji, P Milns, A Murray, D Nicol, T Pinkney, S Ramcharam, T J Royle, R Scarpinata, C Smart, S Smart, N Suggett, S Sutherland, Y H M Tam, A Torrance, M Wall, A Wilkey (Queen Elizabeth Hospital Birmingham); S Vakis* (Queen’s Hospital, Burton); M Bassuini,* J Garner, S Maz, M Mottahedeh,* M Smith, M Withers (Rotherham Hospital); R Krishnamourthy, M Paraoan,* P Thomas (Royal Albert Edward Infirmary); N Chesshire, S Davies, S Gold, I Hamzah, N Hurst, C K Khoo, S Kiani, S Liptrot, J Lund,* C Millard, C Morris, J R Reynolds, I Selvaraj, A Simpson, W Speake, S Tou (Royal Derby Hospital); W Chambers, A S Gee, A Grice, D Johnston, Q Miller, J Pittman,* D Price, R Telford (Royal Devon and Exeter Hospital, Wonford); E Barrow, C Coldwell, I Crighton, T Raymond, S Richmond, P K Patel* (Royal Lancaster Infirmary); M Cheetham* G Kulemeka (Royal Shrewsbury Hospital); M Carraretto, S Huddart, J Kirk-Bayley,* D Moor, M Scott (Royal Surrey County Hospital); M Asif, R Awan, R Bethune, M Chadwick, T Cook, E Courtney, S Dalton,* A Lim, M E R Williamson, J Wood (Royal United Hospital Bath); R Scott, G Branagan* (Salisbury District Hospital); J Bellin, D Browning, V Bulso, N Carter, N Cruikshank, K Gill, N Hulme, E Leno, D Newbould, V Thumbe, H Joy,* K Sankar, S Sivasubramaniam, R Wakhle, R Walton, L Zulueta (Sandwell General Hospital); S M Ahmad, G Kaur,* D Longbottom, M Nnaji, A Ramamoorthy, J Thomas (Scunthorpe General Hospital); I Botteril, D Miskovic,* P M Sagar (St James’s University Hospital); S Bhuptani, R Kennedy, I Jenkins,* S Littler (St Mark’s Hospital); G Arnold, G Buchanan, A Con, P Dawson,* S Lawrie, J Lowe, S Malhotra, P Paraskeva, S Richards, V Sidhu, P Ziprin (St Mary’s Hospital, Paddington); D Kruchek, A Kuttler* (St Peter’s Hospital); K Mohammad, T Nasser, H Rehman, K Siddiqui, A Siddiqui, Q Sindhu, M Soliman, M Solkar,* S Thota (Tameside General Hospital); A Renehan,* C Selvasekar (The Christie); H Melsom, Y J Yiannakou* (University Hospital Of North Durham); A K Agarwal, D Borowski,* F Brohi, D Garg, T Gill, V Mahadevan, A Sharma, M A Tabaqchali (University Hospital Of North Tees); T Alce, A Navapurkar, V Page,* T Stambach (Watford General Hospital); P Capozzi, P Coe, P Conroy, S Duff,* T Grey, O Hill, J Nicholson, D Nolan, J Pollard, A Ramesh, M Sabar, A Sharma, K Telford (Wythenshawe Hospital); D Davies, S Davies,* J Wilson, D Yates (York Hospital).

Ethical approval: The protocol^37^ was approved by the national research ethics committee (NRES No 10/H0402/7) and the Medicines and Healthcare Regulatory Authority (MHRA).

Data sharing: The statistical analysis plan is also available from the study website. Anonymised patient level data will be made available from the corresponding author at the Birmingham Clinical Trials Unit for reasonable requests. Consent was not obtained for data sharing but the presented data are anonymised and risk of identification is low. The final version of the study protocol is available at www.birmingham.ac.uk/Documents/college-mds/trials/bctu/dreams/protocol.pdf

Transparency: The lead author affirms that the manuscript is an honest, accurate, and transparent account of the study being reported; that no important aspects of the study have been omitted; and that any discrepancies from the study as planned (and, if relevant, registered) have been explained.
