# Supplementary material for: Dexamethasone versus standard treatment for postoperative nausea and vomiting in gastrointestinal surgery: randomised controlled trial (DREAMS Trial)
Source: BMJ. 2017 Apr 18;357:j1455. doi: 10.1136/bmj.j1455 (PMC5482348; doi:10.1136/bmj.j1455)
Supplement: Supplementary file 1 — Appendix 1: Supplementary figures A and B [file magl035972.ww1.pdf]

Appendix: Supplementary figures [posted as supplied by author]

Fig A PONV severity of nausea scale

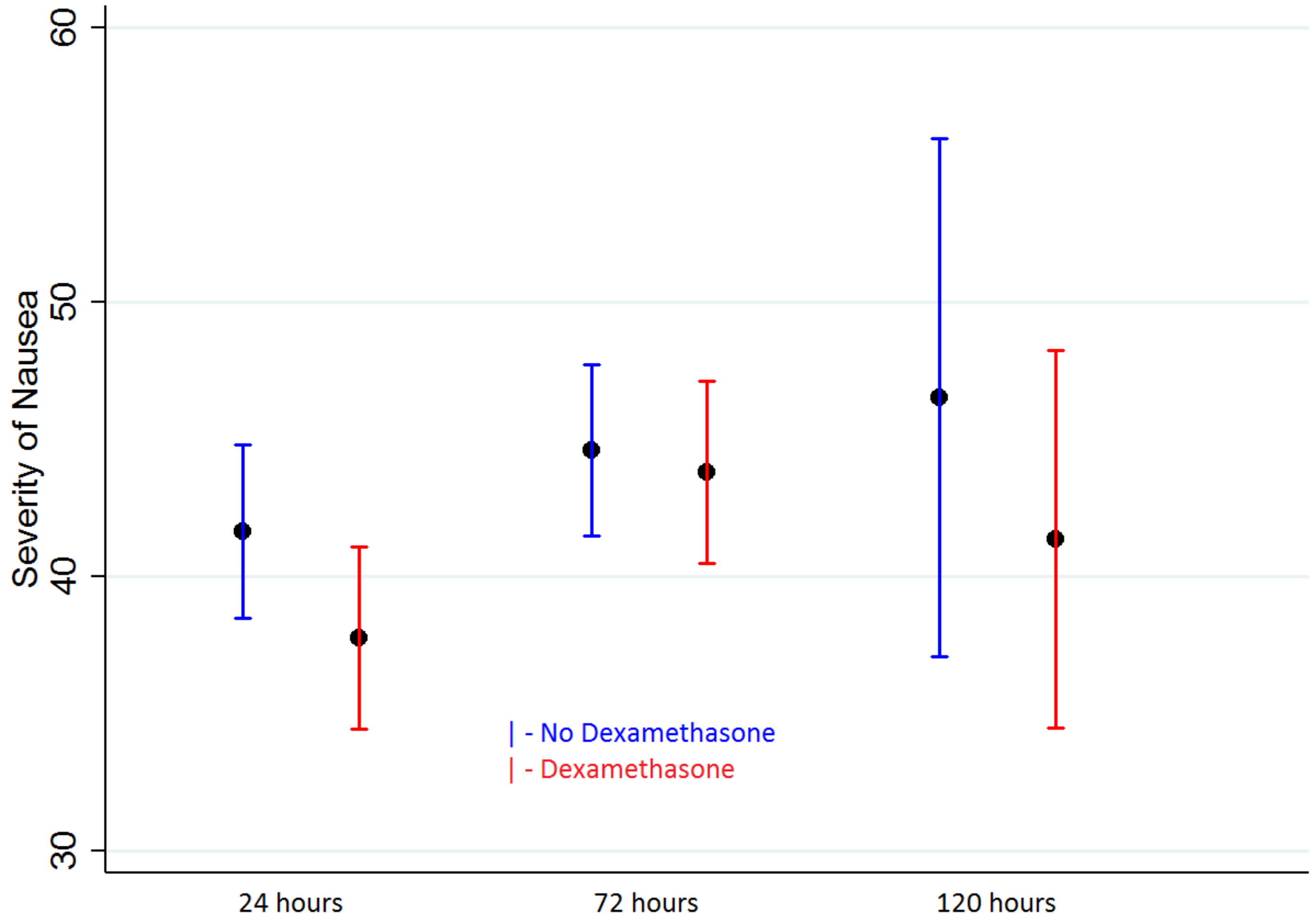

Plotted values are mean VAS scores (high scores indicate high more severe nausea). Error bars depict 95% confidence intervals for the mean.

Fig B Kaplan Meier plot of time to discharge

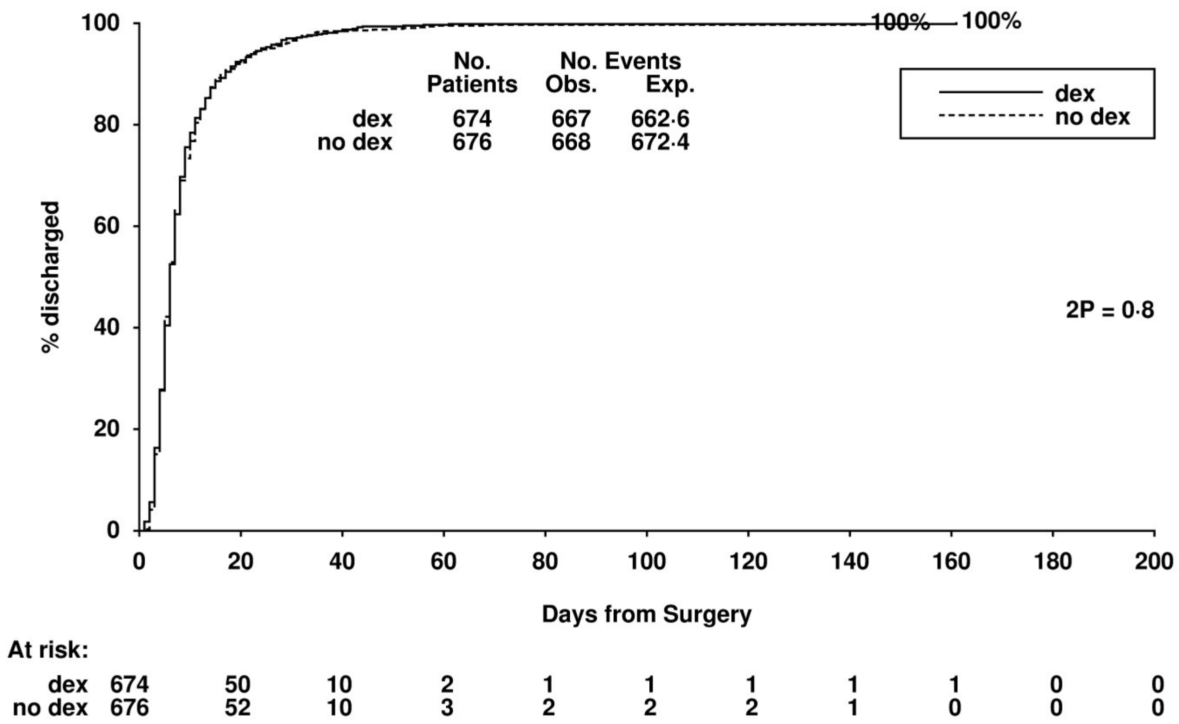

Hazard ratio (dex vs no dex) 1.02 (0.90, 1.14), p=0.79
